# Voxel and surface based whole brain analysis shows reading skill associated grey matter abnormalities in dyslexia

**DOI:** 10.1038/s41598-021-89317-x

**Published:** 2021-05-25

**Authors:** Teija Kujala, Aleksi J. Sihvonen, Anja Thiede, Peter Palo-oja, Paula Virtala, Jussi Numminen, Marja Laasonen

**Affiliations:** 1 Cognitive Brain Research Unit, Department of Psychology and Logopedics, Faculty of Medicine, University of Helsinki, Haartmaninkatu 3 B, P.O. Box 21, 00014 Helsinki, Finland; 2 Department of Neurosciences, Faculty of Medicine, University of Helsinki, Helsinki, Finland; 3 Department of Radiology, Töölö Hospital, Helsinki University Central Hospital, Helsinki, Finland; 4 Department of Psychology and Logopedics, Faculty of Medicine, University of Helsinki, Helsinki, Finland; 5 Department of Phoniatrics, Helsinki University Hospital, Helsinki, Finland; 6 School of Humanities, Philosophical Faculty, University of Eastern Finland, Joensuu, Finland

**Keywords:** Neuroscience, Psychology, Anatomy, Neurology

## Abstract

Developmental dyslexia (DD) is the most prevalent neurodevelopmental disorder with a substantial negative influence on the individual’s academic achievement and career. Research on its neuroanatomical origins has continued for half a century, yielding, however, inconsistent results, lowered total brain volume being the most consistent finding. We set out to evaluate the grey matter (GM) volume and cortical abnormalities in adult dyslexic individuals, employing a combination of whole-brain voxel- and surface-based morphometry following current recommendations on analysis approaches, coupled with rigorous neuropsychological testing. Whilst controlling for age, sex, total intracranial volume, and performance IQ, we found both decreased GM volume and cortical thickness in the left insula in participants with DD. Moreover, they had decreased GM volume in left superior temporal gyrus, putamen, globus pallidus, and parahippocampal gyrus. Higher GM volumes and cortical thickness in these areas correlated with better reading and phonological skills, deficits of which are pivotal to DD. Crucially, total brain volume did not influence our results, since it did not differ between the groups. Our findings demonstrating abnormalities in brain areas in individuals with DD, which previously were associated with phonological processing, are compatible with the leading hypotheses on the neurocognitive origins of DD.

## Introduction

Developmental dyslexia (DD) is a reading-skill impairment with a strong and multifactorial genetic component^1^, which may emerge irrespective of adequate intelligence and reading instruction^2^. It is the most common neurodevelopmental disorder having a prevalence reported to range between 5–17.5%^3^ and 5–10%^4^. Due to being common and having a devastating influence on the individual’s academic achievements, career, and coping^5^ make it pertinent to understand its neural basis. Yet, this task is very challenging due to the heterogeneity of its phenotype^6,7^ and the complexity of the neural network underlying reading^8,9^. According to current leading theories, DD is primarily based on phonological deficits^4,10^ and associated with significant implicit learning problems^11^, and working-memory dysfunctions^12^.

The endeavor to find anomalies in the neural reading circuitry in DD has continued for over 50 years, yet with relatively few replicated results on the neuroanatomical abnormalities in DD and their association with reading-related skills (e.g.^13,14^). Meta-analyses summarizing the heterogenous voxel-based morphometry (VBM) findings have reported grey matter (GM) anomalies mainly in the left occipito-temporal and bilateral superior temporal and parietal areas as well as the cerebellum bilaterally^15–17^. The most recent meta-analysis, including 1164 participants across 18 studies, concluded, however, that even large-scale studies highlight a range of inconsistencies and limitations^14^. Furthermore, according to this analysis the most robust finding in DD is reduced total brain volume, rendering the cortical anomalies specifically associated with DD unsettled.

Besides VBM, a promising approach for searching more subtle neuroanatomical markers^18^ is surface-based morphometry (SBM), which has been, however, scarcely used in DD research. Of the few studies carried out so far, a region of interest analysis found diminished cortical areas in adults with DD in inferior frontal and fusiform regions and abnormal cortical thickness lateralization in the supramarginal area^19^. However, these findings could not be replicated in studies with larger sample sizes^20, 21^.

In addition to the unusually challenging complex geno- and phenotypes of DD, a range of methodological issues have led to a lack of consensus on the GM anomalies in DD and their contribution to DD symptoms. The variation in preprocessing methods, statistical thresholding, and study populations as well as the lack of consistency in adjusting the analyses for confounding effects, like brain size, across the studies may partly explain this, and has given rise to methodological recommendations for more reliable research^14^. On this account, we set out to evaluate the critical GM volume and cortical surface abnormalities in adults with DD, employing neuropsychological testing of functions vital for reading and a combination of up-to-date whole-brain VBM^22^ and SBM^23^, using recommended methods, statistical thresholding, and systematically controlling for relevant covariates. VBM and SBM were chosen (i) to evaluate different levels of GM anomalies in dyslexia, (ii) to complement each other, and (iii) aim to overcome the methodological limitations involved in either of the methods used alone^24^. In addition, whole-brain data-driven analyses were deliberately chosen given the lack of consensus over grey matter anomalies in dyslexia^14^. Based on data discussed above, we expected to find GM anomalies in DD in left reading-related network, and their association with skills essential for reading. Due to lack of consistent results in the few existing SBM studies on DD, no specific hypotheses could be made, but we expected the cortical SBM and VBM findings to overlap.

## Materials and methods

### Participants

Forty-five right-handed Finnish-speaking participants completed the MR imaging, the final sample consisting of 22 typically reading and 23 dyslexic participants with no history of neurological or psychiatric diseases. The groups were balanced in age, years of education and music education, and sex (Table 1), but significantly differed in the reading-skill measures and composite scores of phonological processing, reading skills, and working memory (Table 2). However, they differed in all IQ indices. Verbal IQ (VIQ), but not performance IQ (PIQ), is expected to be lower than normal in DD and, therefore, PIQ was used as a covariate. No group differences were found in total GM, white matter (WM), CSF, total intracranial volume (TIV), or total brain volume (Table 1).

**Table 1:** Demographic and morphological data.

	Dyslexic (*n* = 23)	Control (*n* = 22)	*P* value	*Effect size*
**Demographic**				
Gender (male/female)	11/12	10/12	1.000 (*χ*^2^)	0.02 (V)
Age (years)	31.3 (8.6)	29.8 (5.9)	0.530 (*t*)	0.20 (*d*)
Education (years)	15.7 (5.2)	16.1 (4.4)	0.817 (*t*)	0.08 (*d*)
Musical education (years)	3.0 (7.8)	3.7 (5.5)	0.730 (*t*)	0.10 (*d*)
**Morphological**				
Grey matter volume (litres)	0.76 (0.1)	0.77 (0.1)	0.547 (*t*)	0.18 (*d*)
White matter volume (litres)	0.48 (0.1)	0.49 (0.1)	0.644 (*t*)	0.14 (*d*)
Cerebrospinal fluid volume (litres)	0.27 (0.1)	0.30 (0.1)	0.168 (*t*)	0.41 (*d*)
Total intracranial volume (litres)	1.51 (0.2)	1.56 (0.2)	0.324 (*t*)	0.29 (*d*)
Total brain volume (litres)	1.24 (0.1)	1.26 (0.1)	0.555 (*t*)	0.18 (*d*)

Group sizes (n) and mean values of background variables in the Dyslexic and Control groups with standard deviation in parentheses. P-values show Chi Squared (χ^2^) and independent-samples t-test (t) statistics for group comparisons. Effect sizes show Cohen’s d and Cramer’s V for group comparisons.

**Table 2:** Neuropsychological data.

Neuropsychological composites (bold) and individual tests	Median (IQR)		Range	*p*_*corr*_	Effect size (*r*)
	Dyslexic (*n* = 23)	Control (*n* = 22)			
**Phonological processing [z]**	− 0.2 (1.2)	0.4 (0.4)	[− 2.8 to 1.3]	< 0.000	0.62
Pig Latin (accuracy, amount of correct items out of 15)^1^	9.0 (7.0)	15.0 (1.0)	[0.0–15.0]	< 0.000	0.58
Nonword span length (accuracy, amount of correctly recalled words out of 35)^2^	12.0 (3.0)	13.0 (4.0)	[3.0–19.0]	0.083	0.26
Rapid Alternate Stimulus naming (RAS) (speed of second trial, seconds)^3^	30.0 (10.7)	24.0 (6.4)	[19.7–68.8]	< 0.000	0.60
**Reading [z]**	− 0.3 (0.9)	0.6 (0.2)	[− 3.6 to 0.8]	< 0.000	0.85
Word list reading (accuracy, amount of correctly read words out of 30)	30.0 (1.0)	30.0 (0.0)	[25.0–30.0]	0.005	0.43
Word list reading (speed, seconds to read 30 words)	31.0 (11.4)	19.3 (2.9)	[14.5–83.5]	< 0.000	0.78
Pseudoword list reading (accuracy, amount of correctly read words out of 30)	21.0 (8.5)	28.5 (3.5)	[6.0–30.0]	< 0.000	0.72
Pseudoword list reading (speed, seconds to read 30 words)	72.9 (32.6)	40.1 (7.7)	[31.7–231.8]	< 0.000	0.84
Text reading (accuracy, % of correctly read words in 3 min)^#^	98.2 (1.1)	99.4 (0.8)	[92.4–100.0]	< 0.000	0.60
Text reading (speed, amount of correctly read words in 3 min)^#^	305.0 (67.0)	449.0 (62.8)	[205.0–479.0]	< 0.000	0.82
**Full intelligence quotient**	104.5 (17.3)	118.0 (11.7)	[90.5–130.5]	< 0.000	0.60
Verbal IQ [Wechsler Adult Intelligent Scale (WAIS)-IV Similarities and Vocabulary]	103.0 (20.0)	115.0 (10.0)	[75.0–128.0]	< 0.000	0.57
Performance IQ (WAIS-IV Block design and Matrix reasoning)	113.0 (11.0)	120.5 (11.6)	[81.0–138.0]	0.004	0.45
**Working memory functions**	19.0 (7.5)	24.0 (5.8)	[13.0–32.0]	0.007	0.41
WMS-III Letter-Number Sequencing	10.0 (3.5)	13.0 (3.8)	[7.0–19.0]	< 0.000	0.56
WMS-III Spatial span	9.0 (5.0)	10.5 (3.0)	[4.0–19.0]	0.193	0.20

Notes. Group sizes (n) and median values of all variables in the Dyslexic and Control groups with interquartile range (IQR) in parentheses. Group differences were tested with Wilcoxon sign-rank test, p-values are FDR-corrected, and effect sizes (r) are Wilcoxon Effect Sizes. Composite scores of the test results (bolded) were formed for phonological processing and technical reading by converting the raw scores (of subtests below the respective composite) to z-scores and averaging them, and for working memory according to WMS-III (Wechsler, 2008). For all IQ scores, normalized mean = 100 and SD = 15. For WMS-III subtests, normalized mean = 10 and SD = 3. For WMS-III working memory index, normalized mean = 20 and SD = 6.

^1^Phonological awareness.

^2^Phonological short-term memory.

^3^Rapid serial naming.

^#^Not included in the reading composite score.

A participant was classified as dyslexic if either a recent statement on dyslexia diagnosis was available from a health-care professional (e.g., psychologist), or he/she had reading-related problems in childhood based on the Adult Reading History Questionnaire (ARHQ; cut-off at 43% for the childhood-related items;^25^), confirmed in a clinical interview, combined with a performance of at least one standard deviation (SD) below the average of age-matched standardized control data^26^ in at least two reading subtests (word list reading, pseudoword list reading, text reading) in speed or accuracy (Table 2). Control-group participants (1) had no language-related problems and neither did their parents nor siblings, (2) reported no childhood problems in reading or writing in ARHQ or interview, and (3) performed within norm in at least two out of three reading subtests in both speed and accuracy.

The exclusion criteria were as follows (self-reported in questionnaires and clinical interview except for IQ, which was tested): attention deficit evaluated with the Adult ADHD Self-Report Scale ASRS-v1.1 questionnaire^27^, developmental or other language impairment, other neurological or psychiatric disorders, substance abuse, medication affecting the brain, uncorrected visual deficit, an individualized school curriculum, early bilingualism, PIQ below 80, and non-detachable metal in the body or pregnancy. The study, performed according to the Declaration of Helsinki, was approved by the Coordinating Ethics Committee of The Hospital District of Helsinki and Uusimaa. A signed informed consent was obtained from all participants.

### Neuropsychological tests

The neuropsychological test battery assessed IQ, working memory functions, reading, and phonological processing, combined into four composite scores. They were averages over the z-transformed test scores for reading and phonological processing, and averages of the standardized test scores according to the Working Memory Index in Wechsler Memory Scale (WMS-III) for working memory and according to PIQ, VIQ, and full IQ in Wechsler Adult Intelligent Scale (WAIS-IV) for IQ (Table 2). Reading skills (accuracy and speed; Cronbach’s α = 0.87) were assessed with word and pseudoword list reading tests^28^. The phonological processing composite (Cronbach’s α = 0.69) included ‘Pig Latin’^28^, non-word span length^29^, and rapid alternating stimulus naming^30^, measuring phonological awareness, phonological short-term memory, and rapid access of phonological information, respectively^31^. Working memory functions were evaluated with WMS-III subtests letter-number sequencing and spatial span^32^. Verbal IQ was assessed with WAIS-IV subtests similarities and vocabulary and performance IQ with subtests block design and matrix reasoning.

In the analyses, composite scores were used instead of the individual single-task variables to reduce the number of analyses and the error variance related to single task performance. Due to the data size no factor analysis could be run using, therefore, the classifications based on previous theoretical and factor-analytic studies^31^ and checking the internal consistency of our domain variables with Cronbach’s α (see above).

### MRI data acquisition

A 3 T MAGNETOM Skyra MRI scanner (Siemens Healthcare, Germany) with a 32-channel head coil (AMI center, Aalto University, Espoo, Finland; duration 30 min) was used. High-resolution magnetization prepared rapid acquisition gradient-recalled T1 images were obtained (176 slices, slice thickness 1 mm, flip angle = 7°, TR = 2530 ms, TE = 3.3 ms, voxel size = 1.0 × 1.0 × 1.0 mm^3^). A physician checked the MRIs for incidental findings.

### Voxel-based morphometry

Morphometric analysis was carried out using the Statistical Parametric Mapping (SPM12, Wellcome Department of Cognitive Neurology, UCL) under MATLAB 9.4.0 (The MathWorks Inc., Natick, MA, USA, version R2018a). After reorienting the T1 images using the anterior commissure as origin, the new segmentation algorithm with default parameters, except affine regularization set to the International Consortium for Brain Mapping (ICBM) template for the brains of European participants, was applied to the T1 images, segmenting them precisely into GM, WM, and CSF probability maps. Tissue probability maps were then normalized to the Montreal Neurological Institute (MNI) space using the Diffeomorphic Anatomical Registration Through Exponentiated Lie Algebra (DARTEL) registration process implemented in SPM12. During the process, the imaging data were resampled to 1.5 × 1.5 × 1.5 mm^3^ voxel size and modulated, allowing evaluation of regional volumetric differences. Images were smoothed with an isotropic Gaussian kernel of 8 mm full width at half maximum (FMWH). During each step, the images were visually checked for potential segmentation and registration errors. The TIV was calculated by combining the GM, WM, and CSF images generated during the segmentation.

### Surface-based morphometry

Brain-surface group differences were analyzed using the CAT12 toolbox (C. Gaser, Structural Brain Mapping Group, Jena University Hospital, Jena, Germany; http://dbm.neuro.uni-jena.de/cat/) under SPM12. Default parameters in standard-protocol accordance (http://www.neuro.uni-jena.de/cat12/CAT12-Manual.pdf) were used in segmentation, surface estimation, data resampling, and smoothing. Extracted surface parameters included thickness, gyrification measuring surface complexity in 3D^33^, sulcus depth, and cortical complexity (fractal dimension^34^). As recommended, smoothing filter size in FWHM was 15 mm for thickness data and 20 mm for folding data (e.g. gyrification). The surface data were visually inspected for artefacts and homogeneity and the overall image quality was checked in statistical quality control.

### Statistical analyses

In VBM analysis, one independent-sample t-test with two different contrasts (Controls > Dyslexics, Dyslexics > Controls) was calculated. The results were thresholded using the “Threshold and transform spmT-maps” function in CAT12 toolbox at a default cluster-forming threshold (uncorrected *p* < 0.001) and a familywise error rate (FWE) corrected *p* < 0.05 at the cluster level (alpha-level) and corrected for non-isotropic smoothness^35^. All VBM analyses were adjusted for age, sex, and TIV^36^. In addition, to follow recent recommendations^14^ and to take the group difference into account, PIQ was also added as a covariate of no-interest in the VBM analyses. Neuroanatomical regions were identified using the Automated Anatomical Labeling Atlas^37^ included in the xjView toolbox (http://www.alivelearn.net/xjview/).

In SBM, four independent-samples t-tests (cortical thickness, gyrification, sulcus depth, complexity) with two different contrasts (Controls > Dyslexics, Dyslexics > Controls) were calculated. Like VBM analyses, SBM analyses were thresholded at a default whole-brain threshold (uncorrected *p* < 0.001) and a FWE corrected *p* < 0.05 at the cluster-level and corrected for non-isotropic smoothness. SBM analyses were adjusted for age, sex, and PIQ, but not for TIV as it is not recommended for surface analyses. SBM results were corrected for the total number of carried out surface analyses, that is, alpha-level was set to 0.05/4 = 0.0125.

Partial correlations (two-tailed) were calculated between each individual significant VBM and SBM result and the three composite z-scores (reading score, phonological processing, working memory; Table 2) over the whole sample using SPSS (IBM Corp. Released 2012. IBM SPSS Statistics for Windows, Version 24.0. Armonk, NY: IBM Corp.) whilst controlling for age, sex, TIV, and PIQ. To control for multiple comparisons, false discovery rate (FDR) approach was used and only significant results are reported.

## Results

### Volumetric group differences (VBM)

**Figure 1:**
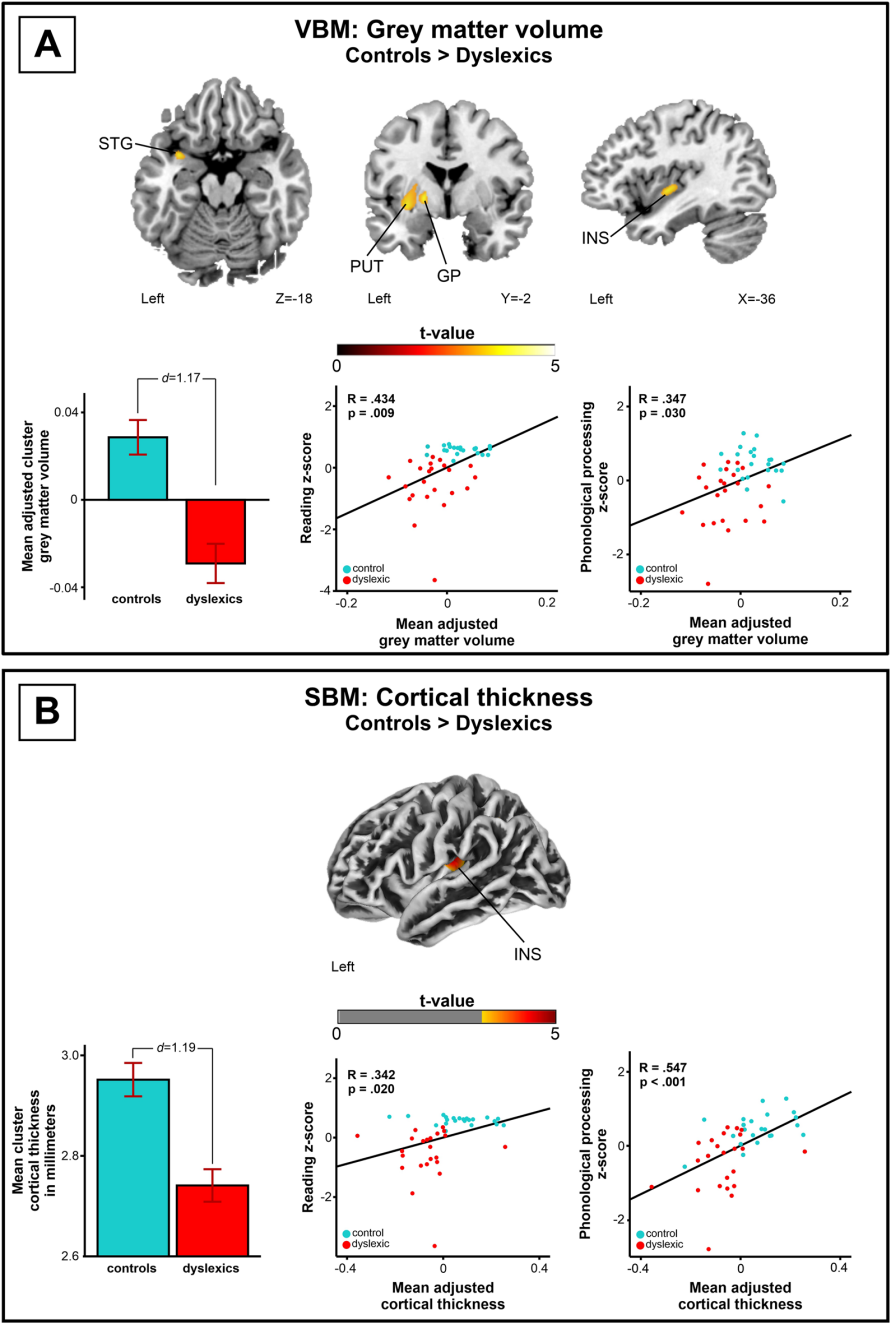
VBM and SBM group differences (see also Table 3). (**A**) Grey matter volume anomalies in dyslexia (Controls > Dyslexics). (**B**) Cortical thickness anomalies in dyslexia (Controls > Dyslexics). N = 45. Statistical maps are thresholded at a cluster-level FWE-corrected *p* < 0.05 threshold. Mean adjusted cluster grey matter volume and mean adjusted cluster cortical thickness correlations to reading-related skills are shown with scatter plots. Bar plots for mean adjusted grey matter volume and mean cortical thickness in significant clusters (Table 3) are shown: bar = mean, error-bar = standard error of mean, *d* Cohen’s d, *GP* globus pallidus, *INS* insula, *PUT* putamen, *STG* superior temporal gyrus.

First, group differences in global brain measurements (total GM, WM, CSF, TIV, and total brain volume) were evaluated with five independent-sample t-tests, and no statistically significant volumetric group differences were found (*p* = 0.168–0.644); see Table 1). In whole-brain VBM analyses, controls had greater GM volume than dyslexic participants in one cluster comprising the left insula, superior temporal gyrus, putamen, globus pallidus, and parahippocampal gyrus. Greater GM volume in this cluster (both groups included) correlated significantly with higher reading (R = 0.434, *p* = 0.009) and phonological processing composite scores (*R* = 0.347, *p* = 0.030; Fig. 1, Table 3).

**Table 3:** Whole-brain VBM and SBM group comparison results.

Method	Contrast	Parameter	Area name	Coordinates	Cluster size	Mean (SD)	p-value	Effect size (d)	Correlations
VBM	Controls > Dyslexics	Grey matter volume	Left Insula (BA 13)	− 33 − 15 2	4266 mm^3^	Controls: 0.029 (0.037) Dyslexics: − 0.029 (0.043) (mean adjusted intensity values)	0.009	1.17	RS: R = 0.434, P = 0.009 PP: R = 0.347, P = 0.030
			Left Globus Pallidus	− 19 − 3 0					
			Left Putamen	− 24 − 1 2					
			Left Superior Temporal Gyrus (BA 38)	− 36 7 − 18					
			Left Parahippocampal Gyrus (BA 34)	− 31 4 − 18					
SBM	Controls > Dyslexics	Cortical thickness	Left Insula (BA 13)	− 35 − 19 − 10	138 vertices	Controls: 2.954 mm (0.148) Dyslexics: 2.739 mm (0.143)	0.008	1.19	RS: R = 0.342, P = 0.020 PP: R = 0.547, P < 0.001

All results are corrected for nonstationarity and thresholded at a cluster-level FWE p < 0.05 threshold.

Correlations are partial correlations with 2-tailed p-value (FDR) controlling for age, sex, TIV and PIQ for VBM, and age, sex, and PIQ for SBM.

*d* Cohen's d, BA, Brodmann area, *PP* phonological processing, *RS* reading score,* SBM* surface-based morphometry, *SD* standard deviation, *VBM* voxel-based morphometry.

### Cortical group differences (SBM)

The control participants had greater cortical thickness in the left insula than the dyslexic participants. Greater thickness in this area (both groups included) correlated significantly with higher reading (*R* = 0.342, *p* = 0.020) and phonological processing composite scores (*R* = 0.547, *p* < 0.001; Fig. 1, Table 3). Gyrification, sulcus depth, and cortical complexity analyses yielded no significant results.

## Discussion

There is an obvious need to understand the neural underpinnings of DD, which is highly prevalent and can have devastating academic, psychosocial, and psychiatric effects on the individual affected (e.g.^5^). Yet, brain abnormalities in DD have remained unsettled due to its heterogenous pheno- and genotypes^1, 7^ and the great methodological variability of previous studies, the most robust finding so far being a lowered total brain volume^14^. By implementing two converging GM analysis methods following up on recent recommendations, combined with careful neuropsychological testing, we compared DD and control samples without total brain volume differences. Furthermore, we determined how reading-related skills are associated with our neuroanatomical findings. Our results showed: (1) diminished GM volume and cortical thickness overlapping in left insula in DD, (2) decreased GM volume in left superior temporal and subcortical areas in DD, and (3) an association between a lower GM volume in all these areas and lower reading and phonological test scores (both groups included in the analysis). Our data pinpoint converging areas for reading-related skills and GM abnormalities in DD in the absence of significant total brain volume differences between the studied groups. This suggests that the occurrence of DD does not (only) rely on brain volume reduction as a predisposing factor or as a de rigueur developmental consequence (see also^19^).

The GM anomalies in our DD sample originated in the left hemisphere where the neural network involved in reading is preponderant^8, 9^. Also, the most consistent functional and structural abnormalities in DD have been found in the left hemisphere^15–17, 38–40^, although they are not limited to it^15^. Our cortical GM volume reduction findings in participants with DD comprised a cluster including superior temporal and insular areas. The involvement of superior temporal areas in reading-related tasks and their lower activation in such tasks as well as diminished volumes in DD have been frequently reported (e.g.^16, 40^). However, the exact area identified by different studies varies, including superior, middle, and inferior temporal gyri, as well as superior temporal sulcus (e.g.^15–17, 41–43^). The present study revealed GM volume reductions in DD in the left superior temporal pole in which previous studies have shown both functional^44^ and structural^41^ anomalies in participants with DD. The left temporal pole is connected with left inferior frontal areas via left uncinate fasciculus, which has previously been implicated in dyslexia^45^, potentially belonging to the temporal-frontal network proposed to underlie the phonological access deficits in DD^10, 46^.

Additionally, our study pinpointed the role of left insula in DD, GM abnormalities in which we found with two complementary methods (VBM, SBM). Previous studies showing structural anomalies in DD in insula are rare^47^ and lack evaluation of the relationship between reading skills and brain structures. The scarcity of previous structural anomaly findings in the left insula in DD might owe partially to the lack of systematical use of relevant covariates. Here, in addition to age and gender, the analyses were controlled for PIQ and brain volume differences (VBM), both of which have been shown to affect volumes of brain regions, including the insula^48, 49^. Consistent with our results, a recent analysis on functional brain networks identified the left insula as a critical hub in DD^50^. Insula is highly connected with the adjacent fronto-temporal, parietal, and subcortical regions, including anterior and posterior language areas^51, 52^. Left insula has an important mediating role in speech production^53^ and phonological processing^54, 55^, and its posterior part is particularly active in the post-articulatory period during both reading and naming^56^. Moreover, consistent with our results, insular dysfunctions have been uncovered in individuals having DD and a phonological deficit^57^. It was also shown to underlie deficient temporal processing of speech and non-speech sounds in DD^58^. Left insula in DD also shares fewer connections with other nodes in a left-hemispheric reading network comprising temporo-parietal and occipital regions^59^. This is compatible with the suggestion that DD might be a disconnection syndrome, with poor neural communication between key brain areas involved in reading and, therefore, vitally contributing to this disorder^54, 60^. Moreover, evidence from lesion studies suggests that damage to the left insula underpins acquired dyslexia^61^. Whereas previous studies reporting left insular structural anomalies in DD are scarce, taken together, these findings suggest that the left insula plays a role in reading, and its structural and functional anomalies in DD should be confirmed and explored further in future studies.

Subcortical structures, so far scarcely studied in DD, have recently been proposed to have a role in this and related developmental language disorders^11, 62^. We found diminished GM volume in DD in left striatum (globus pallidus, putamen) and parahippocampal gyrus. Corticostriatal and hippocampal learning systems are implicated in language and procedural learning, impairments of which have been associated with reading deficits^11, 62^. Consistent with our results, few previous studies have revealed GM anomalies in the left striatum in DD^41, 55^. It has been shown that the connectivity between the left striatum and insula are important in reading, especially in children, suggesting its essential role in early reading acquisition^63^. Moreover, the connectivity between left striatum and insula is altered in DD and left striatum (putamen) has been suggested to contribute to phonological dysfunctions in DD^55^.

Neuroanatomical studies on DD combining VBM and SBM are so far scarce. While VBM has remained as one of the most widely used automatic computational neuroanatomy techniques, it has its own limitations concerning used preprocessing parameters and, for example, sample size, that can contribute to the heterogeneity of previously reported GM findings in DD. Unequal sized groups in a VBM study can produce an inflated false positive rate whereas with groups of equal size (in the present study 22 vs. 23), false positive rate has been shown to be at the expected rate (i.e., about 5%)^64^. However, the interpretation of volumetric GM anomalies in DD, even when following best practices, remains difficult, given that GM volume arises from cortical thickness and area. Here, using both VBM and SBM in concert allows more accurate evaluation of GM anomalies in DD while overcoming limitations involved in either of the methods used alone. The surface-based coordinate system is more accurate than the volumetric one, providing the opportunity to study subtle neuroanatomical anomalies associated with DD^24^. Importantly, the present results revealed overlapping GM volumetric and cortical thickness anomalies in DD in the left insula, suggesting that the decreased cortical thickness gives rise to the observed volumetric anomaly as well. Future studies on DD combining volumetric and surface-based analyses in a large sample of participants with DD are needed as they might reveal other cortical anomalies in DD, for example in gyrification, which the present study failed to find.

The most pertinent issue in studying neuroanatomical anomalies in DD has been the lack of consistency across the reported brain regions. This can be a consequence of the complexity and phenotypic heterogeneity of DD^6, 7^. The most extensive and recent meta-analysis did not find consistent evidence for local GM abnormalities in DD, reporting a reduced total brain volume as the most systematic finding^14^. Lowered total brain volume may result from or be associated with a wide range of confounding issues which could underlie the current inconsistent picture on the neuroanatomical origins of DD. Possibly having groups not significantly differing in total brain volume and controlling for relevant confounding factors at least partly explains our results, which converge with a number of neurofunctional studies on DD, but share only little overlap with previous meta-analytical neuroanatomical reports.

In conclusion, we found GM anomalies in the left superior temporal, insular, and striatal-hippocampal areas in DD. These areas subserve phonological and implicit learning functions, the deficits of which are thought to vitally contribute to DD^4, 11^. Previous anatomical studies linking the structure of these areas with DD is scarce, but especially functional evidence supports our findings. However, future studies with similarly rigorous methodology and groups with matched total brain volumes as here, but including larger participant samples, should further evaluate these brain regions and their contribution to phonological and implicit learning functions in DD as well as their functional and structural connectivity with the reading network. Furthermore, in order to disentangle the effects of inherited factors leading to DD and those caused by this disorder (for example, less exposure to print, atypical reading strategies), longitudinal studies determining brain structure abnormalities prior to and after reading-skill acquisition are needed.

## Data Availability

Anonymized data are available upon reasonable request from the corresponding author.
